# The Photoreaction of the Proton-Pumping Rhodopsin 1 From the Maize Pathogenic Basidiomycete *Ustilago maydis*

**DOI:** 10.3389/fmolb.2022.826990

**Published:** 2022-02-25

**Authors:** Mariafrancesca La Greca, Jheng-Liang Chen, Luiz Schubert, Jacek Kozuch, Tim Berneiser, Ulrich Terpitz, Joachim Heberle, Ramona Schlesinger

**Affiliations:** ^1^ Institute of Experimental Physics, Genetic Biophysics, Freie Universität Berlin, Berlin, Germany; ^2^ Institute of Experimental Physics, Experimental Molecular Biophysics, Freie Universität Berlin, Berlin, Germany; ^3^ Department of Biotechnology and Biophysics, Biocenter, Julius Maximilian University of Würzburg, Würzburg, Germany

**Keywords:** microbial rhodopsin, photocycle, *Ustilago maydis*, overexpression in *Pichia pastoris*, UV/Vis spectroscopy, indole-3-acetic acid, Raman spectroscopy, fungal pathogenicity

## Abstract

Microbial rhodopsins have recently been discovered in pathogenic fungi and have been postulated to be involved in signaling during the course of an infection. Here, we report on the spectroscopic characterization of a light-driven proton pump rhodopsin (*Um*Rh1) from the smut pathogen *Ustilago maydis,* the causative agent of tumors in maize plants. Electrophysiology, time-resolved UV/Vis and vibrational spectroscopy indicate a pH-dependent photocycle. We also characterized the impact of the auxin hormone indole-3-acetic acid that was shown to influence the pump activity of *Um*Rh1 on individual photocycle intermediates. A facile pumping activity test was established of *Um*Rh1 expressed in *Pichia pastoris* cells, for probing proton pumping out of the living yeast cells during illumination. We show similarities and distinct differences to the well-known bacteriorhodopsin from archaea and discuss the putative role of *Um*Rh1 in pathogenesis.

## Introduction

The role of microbial rhodopsins in different organisms, such as bacteria, archaea and eukaryotes (e.g., algae and fungi), is to couple different qualities of light signals to functionalities of the corresponding organism. These retinylic photoreceptors have a long investigation history starting in the sixties with the discovery of bacteriorhodopsin from *Halobacterium salinarum* (*Hs*BR) (Grote et al., 2014), leading these days to the field of optogenetics where rhodopsins are used to remotely trigger cellular responses by light. The natural function of microbial rhodopsin is very diverse (Spudich et al., 2014; Brown, 2014). Notable examples for outward directed proton transport include *Hs*BR (Oesterhelt and Stoeckenius, 1971; Lanyi, 2004) and proteorhodopsin (Beja et al., 2000) and inward proton pumps as xenorhodopsins (Ugalde et al., 2011; Inoue et al., 2016); Shevchenko et al., 2017) and schizorhodopsin (Inoue et al., 2020; Kojima et al., 2020). Models of ion pumps are halorhodopsins (HRs) (Mukohata and Kaji, 1981; Engelhard et al., 2018) and the sodium pump of *Krokinobacter eikastus* (KR2) (Inoue et al., 2013; Skopintsev et al., 2020). Beside pumps, ion channels have been discovered like channelrhodopsins (Sineshchekov et al., 2002; Nagel et al., 2002; Nagel et al., 2003), whose expression in neuronal cells and tissue led to the development of optogenetics. Beside the light-triggered transport of ions, there are also other functions, including sensory tasks (SRs) (Inoue et al., 2014; Chen et al., 2019), as well as switching of enzyme activities (HKRs) (Mukherjee et al., 2019) and many more (Rozenberg et al., 2021).

Besides elucidating the molecular mechanism of the respective rhodopsin, it is worthwhile to address the light-triggered physiological process. In the case of *Hs*BR, outward pumping of protons generates a proton gradient, which is used by a downstream synthase to produce ATP (Oesterhelt and Stoeckenius, 1973). In other cases, the immediate relevance of rhodopsin activation may not be obvious to the host organism. The situation gets even more intricate when a rhodopsin is proposed to be involved in symbiosis or pathogenesis, i.e. the interaction between and among organisms.

Recently, fungal photoreceptors came into focus for their putative role in pathogenesis (Fuller et al., 2015). One of the first biophysically characterized fungal proton-pumping rhodopsin was from *Leptosphaeria maculans* (LR) (Waschuk et al., 2005), a pathogen of the plant *Brassica napus*. The 3D structure of LR was solved (Zabelskii et al., 2021) and the protein was applied to optogenetic experiments (Chow et al., 2010). In the basidiomycete *Ustilago maydis,* the causative organism of smut disease in maize plants, three rhodopsins have been identified: *Um*Rh1, *Um*Rh2 and *Um*Rh3 (Brown, 2004). It has been shown that the *ops3* gene coding for *Um*Rh3 (Adam et al., 2018), which is initially named Um04125 (Ghosh, 2014), is up-regulated during the infection of the maize plant, implicating a role in pathogenesis. The three *Ustilago* rhodopsins have been investigated for their cellular location and regulation during different life conditions of the basidiomycete (Panzer et al., 2019). Electrophysiological investigations by the same authors identified an influence of indole-3-acetic-acid (IAA), solely on the pump activity of *Um*Rh1. This specific reaction to IAA has already been described for *Fusarium fujikuroi*, a rice pathogen expressing two different retinal proteins, OpsA and CarO (Adam et al., 2018). Whereas OpsA is electrophysiologically inactive, only the latter is affected in its proton pumping behavior by IAA.

IAA is a weak organic acid and is the major auxin in plants, which serves as a plant hormone. It is pivotal for the regulation of plant growth and development including cell division, elongation, and differentiation (Kepinski and Leyser, 2003). Furthermore, IAA might be associated with tumor formation after infection with the basidiomycete *Ustilago maydis* (Reineke et al., 2008). Several reports have shown the ability of plant-colonizing bacteria to modulate auxin signaling in plants and the interference of auxins with the host defense system (Spaepen et al., 2007). It is interesting to note that this growth factor has already been identified to be produced in *Ustilago* cells as early as 1952 and was proposed to be involved in pathogenesis (Wolf, 1952). Later, a 20x higher amount of IAA over healthy stalk tissue was identified in young maize tumors (Turian and Hamilton, 1960). Mutagenesis on the IAA synthetic pathway in the basidiomycete exerted no change on tumor formation of the plant, questioning the pro-active role of the fungus-born IAA production for successful infection (Reineke et al., 2008). A thematic publication elaborated on the relevance of IAA considering its role in different aspects of pathogenesis and symbiosis (Fu et al., 2015). Here it was suggested that in the case of the interaction between *Ustilago maydis* and maize, other factors might induce tumor formation. Also, distortions in retinal synthesis in *Ustilago* preventing reconstitution of active rhodopsins, seems not to have an effect on pathogenesis (Estrada et al., 2009).

It was recently shown that acidic environment of the fungus can induce *Um*Rh1 expression via gene regulation executed by the transcriptional factor NRG1, as well hundreds of other genes as WcoI (Sanchez-Arreguin et al., 2021). The latter, when activated by light influences *Um*Rh1 expression (Panzer et al., 2019) implicating that expression of the rhodopsin is both pH and light dependent. Acidic conditions not only influence *Um*Rh1 expression but also enhance activity (Panzer et al., 2019). NRG1 depleted strains show an effect on pathogenicity bringing *Um*Rh1 again into focus as key factor involved in infection. However, several other genes are also regulated by NRG1, which may influence the pathogenicity process.

In this work, we studied the molecular properties of *Um*Rh1 by different biophysical methods with a focus on the influence of pH and IAA, that are potentially involved in pathogenicity, to understand how these external factors alter pumping activity. We applied UV/Vis spectroscopic methods to *Um*Rh1 in detergent and in lipids. To elucidate the structural dynamics of the protein, we characterized the structure of the chromophore retinal by resonance Raman as well as light-induced changes of the entire protein by FTIR difference spectroscopy. We deployed an activity test that probes proton pumping on intact *Pichia pastoris* cells heterologously expressing *Um*Rh1 without further tedious purification. These experiments are complemented by time-resolved electrophysiology on *Um*Rh1 expressed in NG108-15 cells (Panzer et al., 2019). The relevance of our findings is discussed by comparing mechanistic details of *Um*Rh1 to *Hs*BR, which is the model system for proton translocation in retinal proteins. Finally, we frame our results into the context of the role of *Um*Rh1 in pathogenesis based on the current understanding.

## Materials and Methods

### Protein Expression and Purification

The gene coding for *Um*Rh1 with a C-terminal 6xHis tag was amplified from the plasmid pcDNA5/FRT/TO-UmOps1-eYFP (Panzer et al., 2019) with primers 213t_UMOps1-(EcoR1)-Fwd (ATG​GCT​GAA​TTC​ATG​AAC​GTC​GTA​TCC​GAG​CTG​CT) and 215t_UmOps1-His-rev (GCGGCCHCATGATGATGATGATGATGCTGGGTAACGGTGTGCATTTGGGT) and cloned in the yeast expression vector pPIC9K (Invitrogen) between the *Eco*RI*/Not*I sites. The construct was linearized with *Sal*I and electro-transformed (BioRad, MicroPulser) into *Pichia pastoris* SMD1163 cells. First selection was done on histidine-free plates. Hyper resistance expression strains were selected on YPD plates with increasing geneticin concentrations until 4 mg/ml of the antibiotic, following mainly the manufacturer`s instructions (Invitrogen, Multi-Copy *Pichia* Expression Kit). In short: preculturing was done in BMGY medium and diluted 1:4 into BMMY for the main culture. The protein expression was achieved during two culturing days at 30°C and 130 rpm by four feedings of 2.55 ml 500 μM all-*trans*-retinal in methanol into 500 ml BMMY medium. The cells were harvested at 6,000 x g for 10 min and resuspended in breaking buffer (50 mM sodium phosphate, pH 7.4, 1 mM EDTA, 5% glycerol), supplemented with 20 μg/ml PMSF, 10 μg/ml benzamidine and a protease inhibitor tablet (cOmplete ™, Roche). These cells in suspension are then disrupted five times with a Cell Disrupter (Constant Systems, Model TS, 1.1 kW) at a pressure of 2.7 kbar. The cell membrane fragments were sedimented at 186,000 x g for 3 h. The pellet was resuspended in buffer (100 mM NaCl, 20 mM HEPES, pH 7.4) together with 2% of n-dodecyl-β-D-maltoside (DDM) and stirred overnight at 4°C to solubilize the membrane protein. After removing the debris, the *Um*Rh1 protein was purified via Ni-NTA resin in a batch-based method and concentrated afterwards in a 50 kDa MWCO concentrator (Amicon).

### Bacteriorhodopsin Sample Preparation

*Hs*BR was expressed in the *Halobacterium salinarum* strain S9 as described (Stauffer et al., 2020). The purple membrane suspension was diluted in solubilization buffer (100 mM NaCl, 20 mM HEPES, pH 7.4, 3.75% (w/v) octyl-β-D-glucoside) to a final concentration of *Hs*BR of 2.5 mg/ml and solubilized by stirring for 2 days at 4°C. Afterwards, the *Hs*BR suspension was centrifuged at 90,000 x g, 4°C for 1 h to remove insoluble purple membranes. Subsequently, the protein was passed through a size exclusion chromatography column (HiLoad 16/60 Superdex 200 pg) to exchange the buffer to 100 mM NaCl, 20 mM HEPES, pH 7.4, 0.03% DDM.

### Functional Test of Proton Transportation

*P. pastoris* cells expressing *Um*Rh1 were harvested from 500 ml culture. The medium was exchanged to an unbuffered solution (100 mM NaCl, 10 mM MgCl_2_) by washing twice and subsequent centrifugation at 3,000 x g for 5 min, with more than 30 min of incubation between the two steps. Further additional washing steps were done twice to exchange to 100 mM NaCl. The final cell suspension was adjusted to OD_600_ = 10. The pH of the stirred suspension was monitored by a pH meter (InLab Semi-micro, Mettler Toledo, ctd) in the dark or under continuous illumination by yellow light (Schott, ctd. 150 W Halogen lamp with 515 nm long pass and 1,500 nm short pass filters). The protonophore carbonylcyanid-*m*-chlorphenylhydrazon (CCCP) was used to collapse the proton gradient.

### Electrophysiology

NG108-15 cells were heterologously transfected with lipofectamine 2000 (Thermo Fisher) with pcDNA5/FRT/TO-UmOps1-eYFP (Panzer et al., 2019). Media were supplemented with 3 µM all-*trans*-retinal during protein expression. Cells were mechanically detached and seeded in glass coverslips (12 mm) shortly before the patch-clamp experiment.

Patch-clamp experiments were performed in a patch-clamp setup described in detail before (Adam et al., 2018; Panzer et al., 2019) with some modifications. The beam of a 532 nm DPSS laser was coupled into a 400 µm light fiber that was connected to a fiber optic cannula (Thor labs, CFM14L10, Ø400 µm Core, 0.39 NA). Cells were illuminated directly in front of the fiber with an intensity of 15 mWmm^−2^. Pipette solution contained 110 mM NaCl, 10 mM EGTA, 2 mM MgCl_2_, 10 mM HEPES and the bath solution consisted of 140 mM NaCl, 2 mM MgCl_2_, 2 mM CaCl_2_ and 10 mM buffer (potassium hydrogen phthalate, pH 4.0, pH 5.0; MES, pH 6.0; HEPES, pH 7.0, pH 8.0).

### Reconstitution of *Um*Rh1 in Nanodiscs

The mixture of *Um*Rh1, scaffold protein MSP1D1, and lipid 1,2-dimyristoyl-sn-glycero-3-phosphocholine (DMPC) in the ratio of 1:2:110 was incubated at 25°C, 500 rpm for 1 h (Biometra, TCS ThermoShaker). Another 2 h of incubation followed with biobeads (1:1 w/v) (Biorad, SM-2 Adsorbent). Precipitated material was then removed at 20,000 x g for 10 min. The concentrated nanodiscs mixture was afterwards applied to a gel filtration column (Superdex 200 10/300 GL) on Äkta Avant 25. The fractions with *Um*Rh1 reconstituted into nanodiscs were collected.

### Determination of the Extinction Coefficient of *Um*Rh1 by Hydroxylamine Bleaching and UV/Vis Spectroscopy

The protein with an A_530_ of ca 0.2 was exchanged into 100 mM NaCl, 20 mM HEPES, pH 7.4, 0.03% DDM and recorded at room temperature in an UV/Vis spectrometer (Shimadzu UV-2600i). A solution with 10 mM of hydroxylamine was added and the protein was illuminated with a Xenon Lamp for 5 min monitoring the bleaching of the retinal (530 nm) and the increase of the retinal oxime absorption (360 nm; *ε* = 33,600 M^−1^cm^−1^) as mainly described in (Scharf et al., 1992) with subsequent comparison to the UV/Vis spectrum of the untreated *Um*Rh1. The extinction coefficient was then calculated using the formula: (ε_*Um*Rh1_ = A_530_ * *ε*_oximes_/A_360nm_).

### Flash Photolysis in the UV/Vis Range

Time-resolved UV/Vis experiments were performed using a commercial flash photolysis setup equipped with an Nd:YAG laser (Quanta-Ray; Spectra-Physics) (3 mJ/cm^2^/pulse at 532 nm) essentially as described (Resler et al., 2015). Kinetic traces were recorded between 370 and 650 nm with an interval of 10 nm. At each wavelength, 10 laser pulses were averaged to improve the signal-to-noise ratio. Datasets were analyzed by global exponential fitting. For the pH dependency of the M and O state decay (Figure 8B), the respective decay traces were fitted by a single exponential.

Flash photolysis experiments on *Um*Rh1 and *Hs*BR were performed in the following buffer conditions: 100 mM NaCl, 20 mM HEPES, pH 7.4 (0.03% DDM). Flash photolysis experiments at pH 5 as well as pH-dependent measurements of *Um*Rh1 and *Hs*BR were recorded in 100 mM NaCl, 50 mM buffer mixture containing HEPES, MOPS, MES, glycine and citrate (10 mM for each) and 0.03% DDM.

Experiments on indole-3-acetic acid were performed on *Um*Rh1 dissolved in 100 mM NaCl, 100 mM MES, pH 5.5, 0.03% DDM and 20 mM IAA. Since IAA is insoluble in water, a 0.5 M stock solution was prepared in 0.5 N NaOH. The pH was monitored before and after the flash photolysis to exclude any pH shift during the measurement. (The choice of not using the buffer mixture was on account of previous experiments showing an interaction of the citrate with the IAA).

### Resonance Raman Spectroscopy

Raman spectra were recorded using a LABRAM Raman microscope (JobinYvon, Bensheim, Germany) with laser excitations at 647 nm or 457 nm (diode-pumped solid-state lasers from CrystaLaser, Reno, United States or PhotonTec, Berlin, Germany, respectively; at spectral resolutions of ca. 2 or 4 cm^−1^, respectively) focused on the sample using a ×10 microscope objective (Muders et al., 2014). Aqueous samples (100 mM NaCl, 20 mM HEPES, 0.03% DDM, for pH 7.4 or 20 mM NaAc for pH 5.0) were placed in a rotating quartz cuvette (about 2,000 rpm) to avoid photobleaching of the protein. Illumination to drive the protein into a photostationary state was achieved via an LED with an emission maximum at 530 nm. Spectra of neat toluene were recorded in the same spectral ranges for frequency calibration.

### Fourier-Transform Infrared Spectroscopy

Purified and detergent solubilized protein has been washed with a buffer containing 10 mM NaCl, 5 mM MOPS, pH 7.4, or 5 mM sodium acetate, pH 5.0. Several µl of concentrated protein solution have been gently dried onto BaF_2_ windows under a stream of dried air and subsequently rehydrated by placing 5 µL of an 8/2 (w/w) H_2_O/glycerol mixture next to the protein film. A second BaF_2_ and a spacer were used to seal the transmission window. For experiments in D_2_O, a dried protein film has been rehydrated with an 8/2 mixture of D_2_O and perdeuterated glycerol.

All measurements have been carried out using a Bruker Vertex 80v FTIR spectrometer (Bruker Optics, Ettlingen) in transmission mode at a spectral resolution of 4 cm^−1^ (Muders et al., 2014). Light-induced difference spectra were calculated by subtracting the spectra before (ground state) and under continuous green-light (525 nm) illumination (intermediate states). Each spectrum was recorded with 30 scans and automatically repeated multiple times. To achieve a sufficient signal-noise-ratio, 4,500 and 15,000 scans were averaged for pH 7.4 and pH 5.0, respectively.

## Results

### Functional Expression of *Um*Rh1 in *Pichia pastoris* and Activity Assay

**FIGURE 1:**
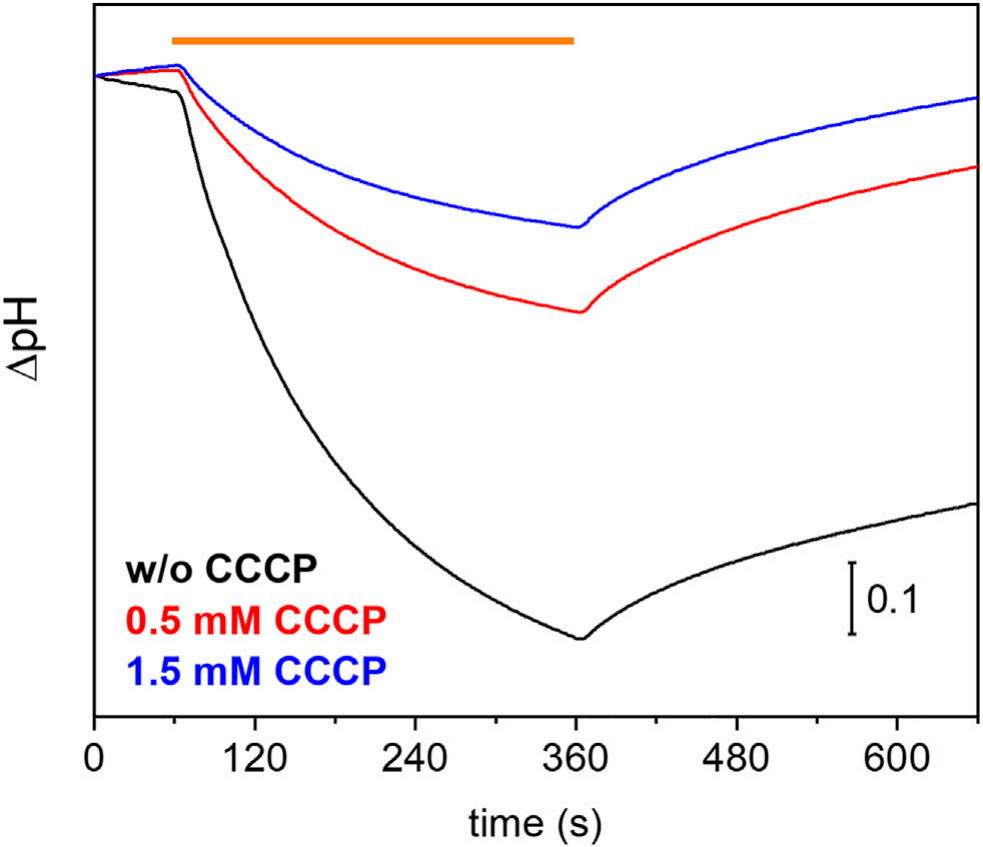
Proton-pumping assay on Pichia cells. Comparison of pH changes during and after 5 min of illumination (indicated by orange bar) of *Um*Rh1 expressing *P. pastoris* cells in 100 mM NaCl in the absence (black trace) and the presence of 0.5 mM (red trace) and 1.5 mM of CCCP (blue trace) as protonophore.

Many eukaryotic microbial rhodopsins, e.g. channelrhodopsins, have been functionally expressed in the yeast *Pichia pastoris* (Bamann et al., 2008) (Muders et al., 2014). In exceptional cases, the eukaryotic microbial rhodopsin was also successfully produced in *E. coli* (Kikuchi M. et al., 2021). Here, we show successful expression of *Um*Rh1 in the yeast cell system with a very high yield (>20 mg/L culture, which corresponds to 5 g of membranes after cell disruption). Previous electrophysiological studies showed that *Um*Rh1 acts as an light-activated outward-directed proton pump (Panzer et al., 2019). Proton pumping activity was increased either in acidic (pH 5) or alkaline (pH 9) conditions compared to activity at nearly neutral conditions (pH 7.4). Functionality of *Um*Rh1 residing in intact *Pichia pastoris* was evaluated by recording the bulk pH of the yeast cell suspension with the help of a pH electrode connected to a pH meter. This method has been widely applied to *E. coli* expression systems as an assay of proton-pumping activity of a variety of different microbial rhodopsins (Sudo et al., 2011). As was recently shown (Kikuchi C. et al., 2021), this approach is transferable to *Pichia pastoris* cells. During continuous illumination with yellow light to activate *Um*Rh1, the pH decreased over time (Figure 1, black trace) indicating active outward proton transport. The transient pH decrease was less upon addition of the protonophore CCCP (Figure 1, red and blue traces), which demonstrates selective proton pumping by *Um*Rh1.

### pH Dependence of Retinal Absorption and Extinction Coefficient of *Um*Rh1

**FIGURE 2:**
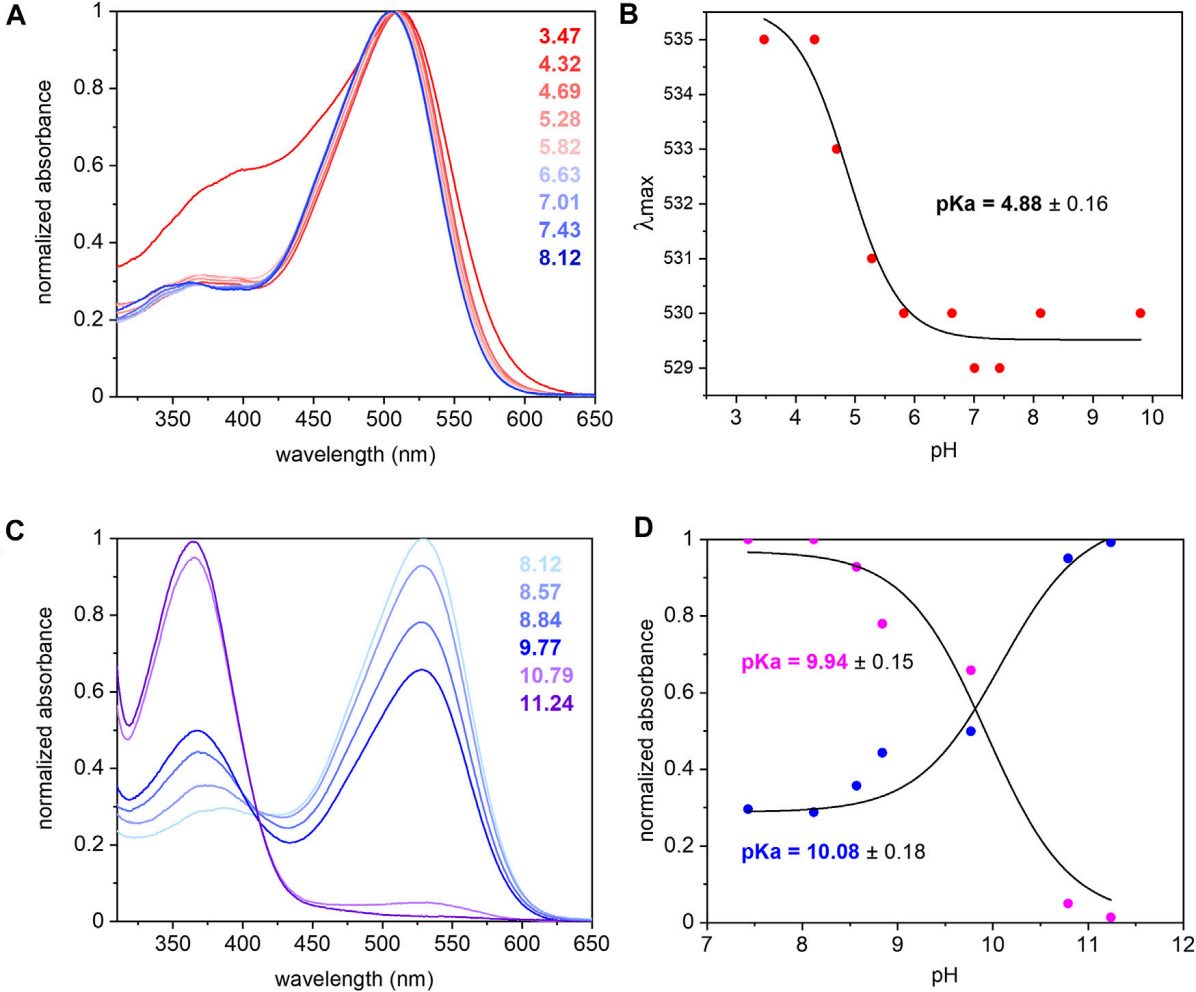
UV/Vis spectra of *Um*Rh1 at different pHs and retinal extraction. *Um*Rh1 was dissolved in an aqueous solution of 100 mM NaCl, 10 mM citrate, 10 mM MES, 10 mM HEPES, 10 mM MOPS, 10 mM glycine, 0.03% DDM, and titrated by addition of small volumes of 1 N NaOH or HCl. **(A)** UV/Vis spectra were recorded at a starting pH of 8.12 and after stepwise addition of 1 N HCl to reduce the pH. **(B)** The maximal absorption of *Um*Rh1 is plotted against pHs and fitted to the Henderson-Hasselbalch equation to derive the pKa of the color transition as described (Inoue et al., 2018). The equation $\lambda_{\max}=\upsilon /\left(1+10\wedge \left(\mathrm{pH}-\mathrm{pKa}\right)\right)+\omega$ is applied,*v* represent the maximum $\lambda_{\max},\omega$ is the offset. **(C)** UV/Vis spectra of *Um*Rh1 in the alkaline range of pH 8.12–11.24. **(D)** The pKa estimation in alkaline range is done by fitting the normalized absorbance at both 530 (magenta) and 365 (blue) nm in different pHs as described in **(A,C).** For the accumulation of blue-shifted state (365 nm), the equation A=υ/(1+10∧(pKa−pH))+ω is used.

The UV/Vis spectrum of purified *Um*Rh1 shows maximal absorption at 530 nm at pH 7.4 (Figure 2A). This absorption peak is red-shifted by 5 nm upon acidification (Figure 2A). From the titration curve (Figure 2B), a transition with a pKa of around 5 has been derived by applying a fit to the Henderson-Hasselbalch equation. This pKa is related to the protonation of an amino acid side chain that electrostatically interacts with the retinal chromophore.

The pKa of the retinal Schiff base is derived by pH titration into the alkaline region (Figure 2C) and determined to be around 10 (Figure 2D) which is significantly lower than in *Hs*BR (pKa = 13.3) residing in the purple membrane of *H. salinarum* (Druckmann et al., 1982).

The extinction coefficient of retinal in *Um*Rh1 was determined by bleaching with hydroxylamine. Using the well-known extinction coefficient of the resulting retinal oxime (*ε* = 33,600 M^−1^cm^−1^) (Scharf et al., 1992) the ratio of the two absorption bands (Supplementary Figure S1) provides the extinction coefficient of 48,000 M^−1^ cm^−1^ for *Um*Rh1.

### Photocycle of *Um*Rh1 at Neutral pH

**FIGURE 3:**
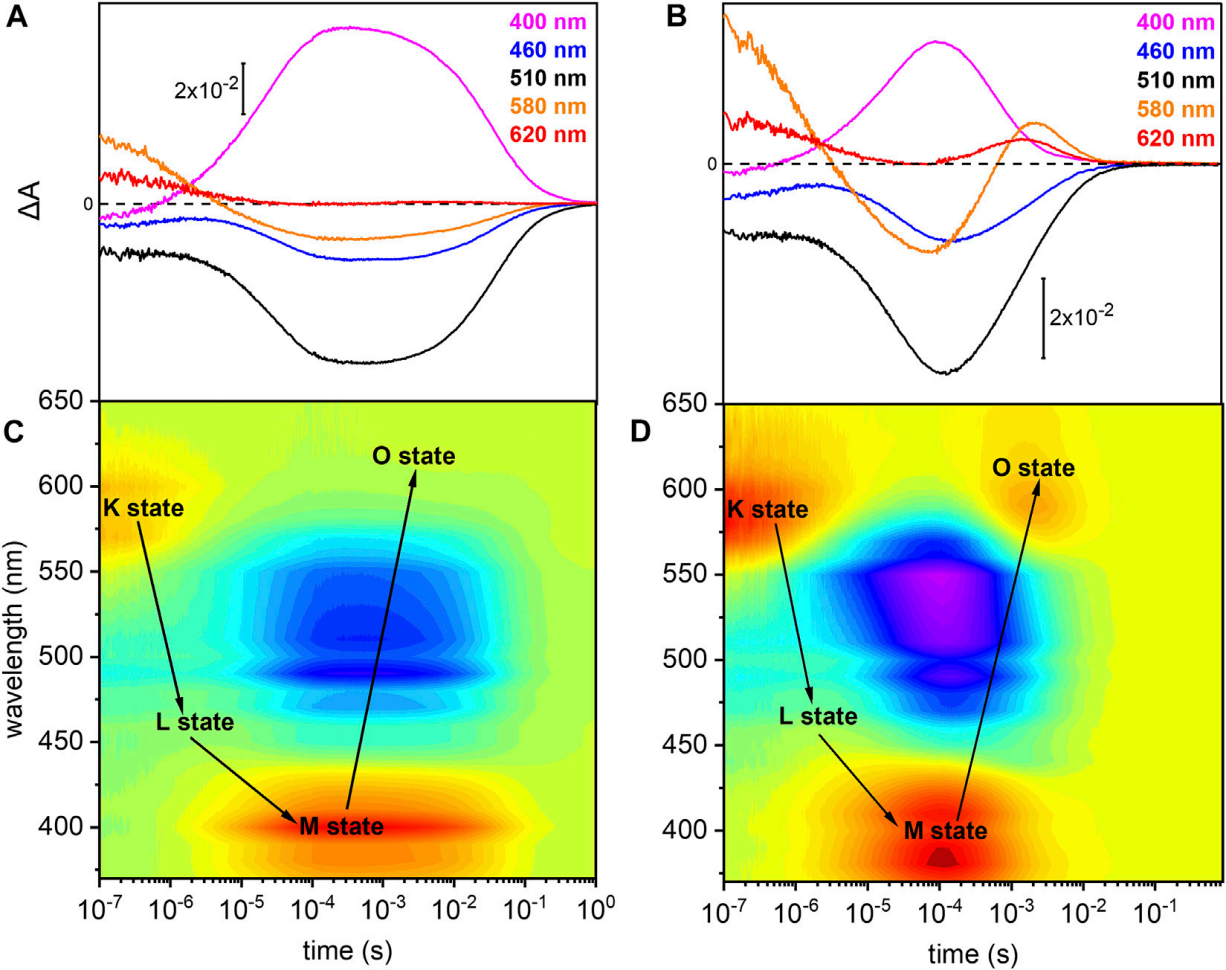
Photoreaction kinetics of *Um*Rh1 recorded at pH 7.4 and pH 5. Time evolution of the flash-induced absorption changes of DDM-solubilized *Um*Rh1 **(A)** in the presence of 100 mM NaCl, 20 mM HEPES, pH 7.4, 0.03% DDM, and **(B)** in the presence of 100 mM NaCl, 50 mM buffer mix (10 mM HEPES, MOPS, MES, citrate and glycine), pH 5, 0.03% DDM. The extracted wavelengths at 400, 460, 510, 580, and 620 nm, are characteristic to M state, L state, ground state, K and O states, respectively. Color maps are showing the absorption changes of *Um*Rh1 at pH 7.4 **(C)** and pH 5 **(D)** over the spectral range of 370–650 nm and the time range of 10 ns–1 s. Red color indicates positive bands from the rise of intermediate states and blue corresponds to the negative absorption change from transient depopulation of ground-state *Um*Rh1. The sequential interconversion between the states is shown with solid arrows.

The photoreaction of detergent solubilized *Um*Rh1 was traced by time-resolved UV/Vis spectroscopy (Figure 3). After pulsed excitation of *Um*Rh1 at pH 7.4, a red-shifted absorption at 580 nm was observed with respect to the ground state (Figure 3A). We termed this intermediate as K state, in analogy to *Hs*BR. In the time range between 1 and 10 µs, another intermediate was detected, with a blue-shifted absorption; this emerging state has a maximum at around 460 nm which corresponds to the L state of *Hs*BR. The L state decayed into the strongly blue-shifted M intermediate, which was observed at 400 nm. The recovery kinetics of the ground state detected at 510 nm match the decay of the M intermediate (*τ* = 40 ms). The O intermediate at 620 nm is hardly discernible at pH 7.4 (Figure 3A). Figure 3C presents the transient absorption changes across the whole range from 370–650 nm in a color map. Positive difference absorbance (yellow to red) reflects the accumulation of the intermediate states K, L, M and O, while the negative difference absorbance (blue) corresponds to the depletion of ground-state *Um*Rh1. The interconversion between the states is shown with solid arrows. The absorption changes due to the L and O states are implicit.

Direct comparison of the kinetics of *Um*Rh1 to the well-known photocycle kinetics of *Hs*BR embedded in its native purple membrane, is not valid as the former was solubilized in the detergent DDM. Therefore, we solubilized *Hs*BR also in DDM and recorded kinetic traces at selected wavelengths (Supplementary Figure S2). It is evident that the decay of the K states is identical in both proteins but rise and decay of the M state are both 4x slower in *Um*Rh1 as compared to *Hs*BR.

### *Um*Rh1 Exhibits a Faster Photocycle at pH 5

Previous patch-clamp experiments have shown that *Um*Rh1 is an outward proton pump, but unlike *Hs*BR, has a different behavior under acidic conditions (Panzer et al., 2019) where *Um*Rh1 exhibits an increased pump activity at pH 5 as compared to neutral and alkaline pH. For these reasons, we examined the photocycle at pH 5 (Figures 3B,D). The recovery of the dark state, monitored at 510 nm, is accelerated as compared to pH 7.4. In acidic environment, a prominent O state is populated during the photocycle. Rise and decay of the O state are faster than for *Hs*BR (red and black traces in Supplementary Figure S3, respectively). Most remarkably, M decay recorded at 400 nm is dramatically accelerated at acidic pH (pink trace in Supplementary Figure S3) and much faster than for *Hs*BR (black trace in Supplementary Figure S3).

### pH Dependence of the Photocycle Kinetics

**FIGURE 4:**
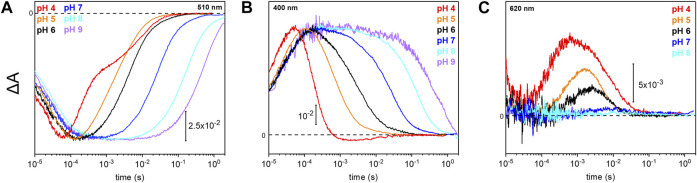
Photoreaction kinetics of *Um*Rh1 at different pH. Temporal evolution of the flash-induced absorption changes of the DDM-solubilized *Um*Rh1 in the presence of 50 mM buffer mix (100 mM NaCl, 10 mM HEPES, MOPS, MES, citrate and glycine, 0.03% DDM), recorded at different pHs. Photoreaction kinetics of the ground-state bleaching at 510 nm in panel **(A)**, M state monitored at 400 nm in panel **(B)** and O state, monitored at 620 nm in panel **(C)**.

Due to the observed changes of the photocycle at neutral and acidic pH, we systematically measured the dependence of the photocycle intermediates in the pH range of 4–9 (Figure 4). Therefore, transient UV/Vis absorption spectroscopy was performed at three different characteristic wavelengths. The ground state recovers slower with increasing pH as retrieved from the kinetic trace recorded at 510 nm (Figure 4A). Intermediates K and L were pH insensitive, whereas the kinetics of the M and O intermediates display massive pH dependences. The rise of the M state, monitored at 400 nm, is largely pH-independent, except for pH 4 where the increase is accelerated. In contrast, the M decay gets stepwise slower with alkalization (Figure 4B) following the trend of the recovery kinetics. The O state, detected at 620 nm, rises faster under acidic conditions (Figure 4C). The O state was only observable at pH 6 and below. At neutral and alkaline pH, the M state dominates at the expense of the O state.

### pH Dependence of Proton Pumping

**FIGURE 5:**
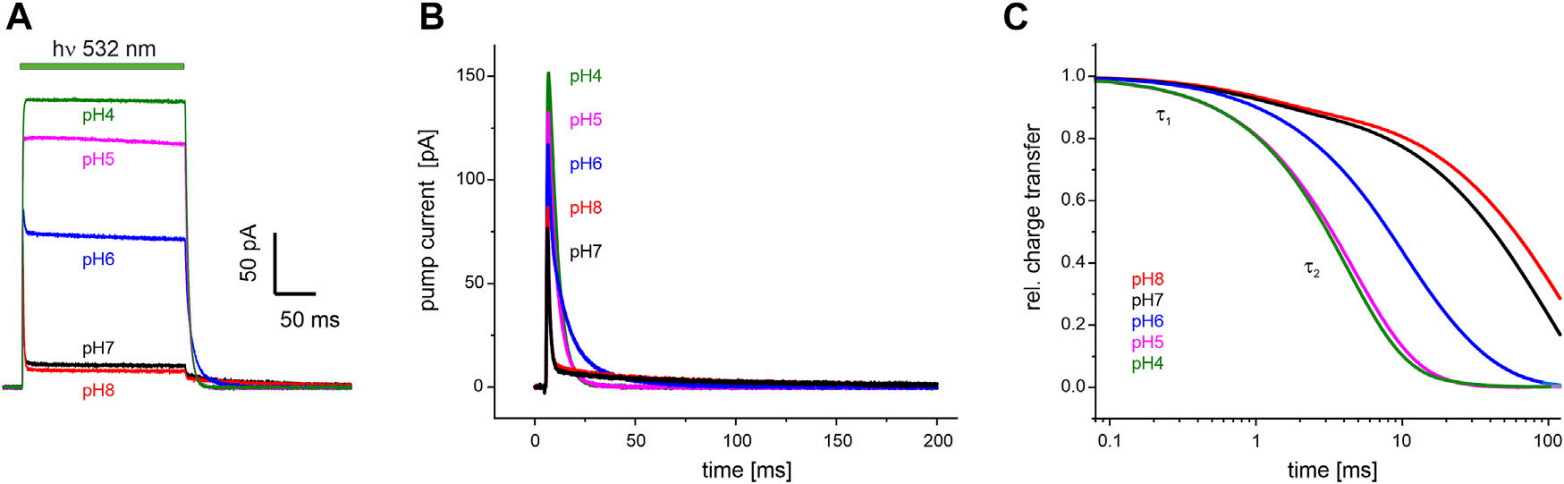
Electrophysiological recordings on *Um*Rh1 at different extracellular pH. Representative traces of whole-cell patch-clamp experiments on NG108-15 cells heterologously expressing *Um*Rh1-eYFP. **(A)** Stationary currents under continuous illumination with a 532 nm DPSS laser at 0 mV command potential and different extracellular pH as indicated. **(B)** Single-turnover measurements of *Um*Rh1 at different pHs recorded after applying a light pulse of < 1 ms. Current amplitudes are highest at acidic pH. At alkaline but not acidic conditions, a pronounced slow component of the current decay is visible. **(C)** Kinetics of charge transfer at different pH plotted on the logarithmic time axis and normalized to the maximum charge recorded at the respective pH. Two exponentials (with τ_1_ and τ_2,_ Table 1) are required to adequately fit the data.

To study how pH affects the pumping activity of *Um*Rh1, we performed patch-clamp experiments on NG108-15 cells expressing *Um*Rh1. We exposed the cells to different extracellular pH in the range from 4–8 and recorded photocurrents under constant illumination (Figure 5A). In accordance with previous finding (Panzer et al., 2019), we noted a strong increase in the stationary photocurrents at acidic conditions.

Single-turnover kinetics of *Um*Rh1 expressed in NG108-15 cells were recorded by time-resolved patch-clamp experiments after pulsed light excitation (Figure 5B). This approach facilitates comparison to our time-resolved UV/Vis experiments (Figure 4) which were also performed with pulsed excitation and avoids potential secondary photoreactions by excitation of intermediate states. As the current amplitude scales with time, we have converted currents into charges (Figure 5C). Kinetics in the pH range between 4-8 have been analyzed by exponential fitting. Evidently, two components are required to adequately fit the data traces with a pH-independent faster time constant (τ_1_) and the slower time constant (τ_2_) displaying strong pH dependence (Table 1).

**TABLE 1:** pH dependence of the decay of the photocurrents of *Um*Rh1. Fitting the decays with the sum of two exponentials (see Figure 5B for a set of representative traces) resulted in the time constants τ_1_ and τ_2_ with the relative deviation derived from five independent experiments recorded under the same conditions.

pH 8	τ_1_ = 0.78 ± 0.25 ms, τ_2_ = 42.1 ± 7.01 ms
pH 7	τ_1_ = 0.77 ± 0.31 ms, τ_2_ = 30.6 ± 8.13 ms
pH 6	τ_1_ = 0.81 ± 0.22 ms, τ_2_ = 12.0 ± 1.44 ms
pH 5	τ_1_ = 0.85 ± 0.29 ms, τ_2_ = 7.85 ± 3.36 ms
pH 4	τ_1_ = 0.74 ± 0.19 ms, τ_2_ = 4.23 ± 0.56 ms

### Photocycle of *Um*Rh1 Reconstituted in Lipid Nanodiscs

As our previous experiments were performed on proteins purified and dissolved in the detergent DDM, we have reconstituted *Um*Rh1 into nanodiscs comprised of DMPC to generate a well-defined lipid bilayer. Time-resolved UV/Vis spectroscopy showed that the kinetics of the K and L photointermediates (Supplementary Figures S4, S5) were not influenced by the presence of the lipid environment. On the ms-s time scale, however, flash photolysis experiments reveal distinct deviations for the decay of the M and O states. As compared to the detergent solubilized protein (Figure 3A), especially the decay of these intermediates slowed down (Supplementary Figure S4). It is worthwhile to note that *Um*Rh1 reconstituted in nanodiscs exhibits the same pH dependency of the photocycle as detergent-solubilized protein (compare Supplementary Figure S5 and Figure 3B). This result indicates that the O state is pH-dependent and predominant at low pH, a behavior that is independent of the membrane environment.

### Influence of Indole-3-Acetic Acid on the Photocycle of *Um*Rh1

**FIGURE 6:**
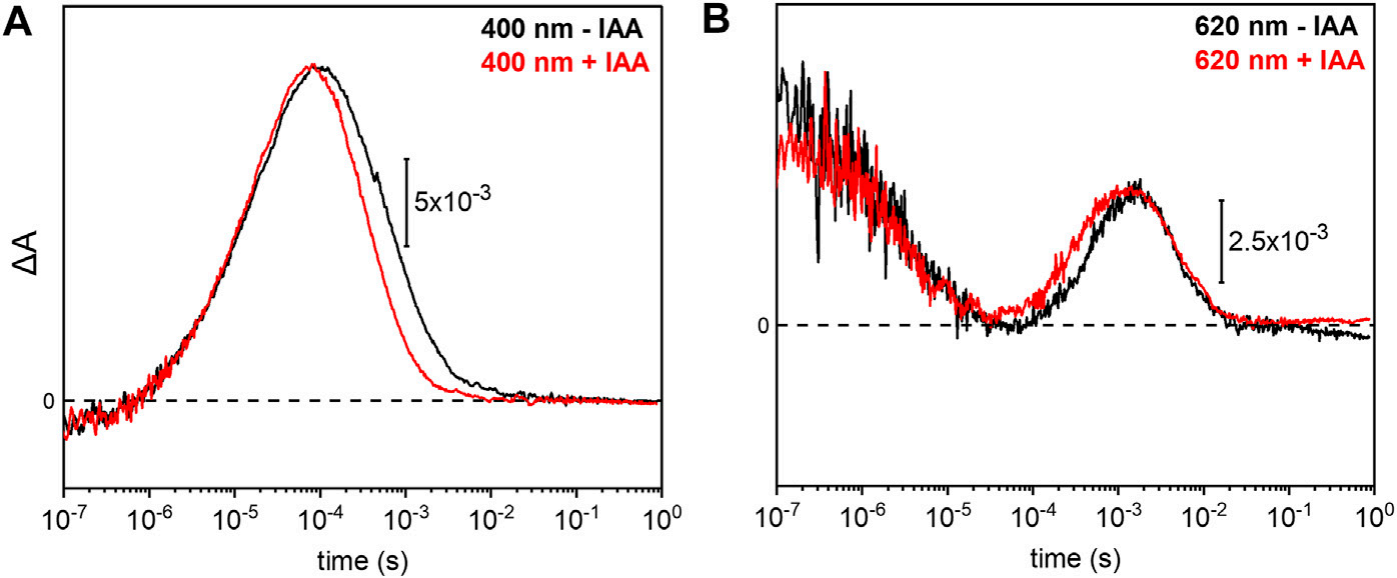
Impact of indole-3-acetic acid (IAA) on the M-O transition of *Um*Rh1. Kinetic traces of the flash-induced absorption changes recorded at **(A)** 400 nm and **(B)** 620 nm of DDM-solubilized *Um*Rh1 (100 mM NaCl, 100 mM MES, pH 5.5 and 0.03% DDM) (black lines) and DDM-solubilized *Um*Rh1 in presence of 20 mM IAA, in the same buffer (red lines), is shown. Great care was taken to perform both measurements at the exact same pH. Experiments have been repeated on three independent samples yielding the same results.

Previous electrophysiological studies revealed that the pump activity of *Um*Rh1 was influenced by the presence of IAA (Panzer et al., 2019). This effect was detected in *Um*Rh1 and other CarO-like rhodopsins (Adam et al., 2018) but rarely observed in other fungal rhodopsins. To probe the molecular effects of IAA, we have compared the photocycle of *Um*Rh1 in acidic conditions in the presence and absence of IAA. It is evident from the kinetic traces that the presence of 20 mM IAA selectively accelerates the M decay (Figure 6A). Correspondingly, the rise of the O state is accelerated as well (Figure 6B). The kinetics of the transitions of other intermediates are not influenced by the presence of IAA (Supplementary Figure S6).

### Vibrational Spectroscopy on *Um*Rh1

**FIGURE 7:**
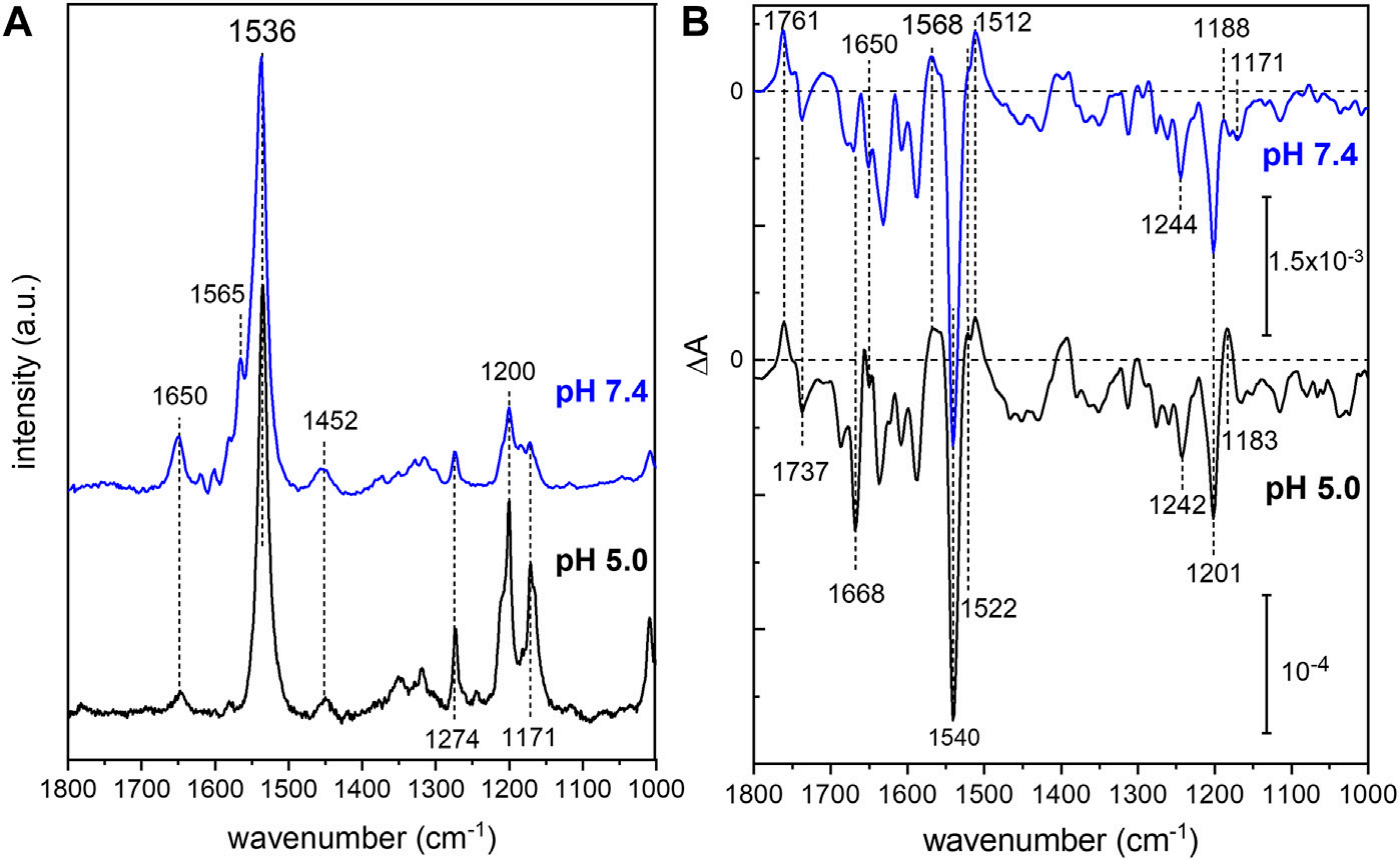
Vibrational spectra of *Um*Rh1. Resonance Raman spectra **(A)** were recorded at 647 nm (pH 5) and 457 nm (pH 7.4). Light-induced FTIR difference spectra **(B)** have been obtained under continuous illumination with a green LED (λ = 525 nm). Blue spectra correspond to pH 7.4 and black spectra to pH 5. The spectrum recorded at pH 7.4 has been scaled to match the signal size of the spectrum recorded at pH 5.

In order to analyze the retinal configuration of *Um*Rh1, Raman spectra were recorded at two different laser excitations, i.e., 647 nm or 457 nm, to achieve different resonant conditions. The 647 nm laser emission is pre-resonant to the ground state, allowing to probe the retinal structure of the ground state of *Um*Rh1 without excitation of the photoreaction (Figure 2A). In turn, the 457 nm laser initiates the photocycle, thus, a photostationary mixture of ground and blue-shifted photointermediates will be probed. Figure 7A shows Raman spectra recorded at 647 and 457 nm excitation at pH 5 and 7.4, respectively. At 647 nm identical spectra are observed at both pH conditions (Supplementary Figure S7) with the most prominent feature, i.e. the ethylenic C=C stretch, located at 1,536 cm^−1^. This frequency is consistent with the all-*trans* retinal configuration, and the highly symmetric line shape indicates that no other configurations are adopted in the ground state (Muders et al., 2014) (Smith et al., 1985). This conclusion is in line with the CC-H in-plane vibration resonating at 1,274 cm^−1^ as well as the most prominent C-C stretches in the fingerprint region at 1,200 and 1,171 cm^−1^ (Muders et al., 2014). The band at 1,452 cm^−1^ is assigned to the CH_3_ deformations of the retinal methyl groups (Alshuth and Stockburger, 1981).

Upon photoexcitation with 530 nm, the O state is accumulated to the photostationary state and the spectrum recorded with 647 nm Raman excitation of the sample at pH 5 is expected to show bands of this red-shifted photocycle intermediate. Extracting these changes in terms of a difference spectrum (light–dark state) (Supplementary Figure S7 trace c) reveals an ethylenic C=C stretch at ca. 1,523 cm^−1^, reminiscent of the O state of *Hs*BR (Smith et al., 1983).

The resonance Raman spectrum recorded with 457 nm excitation (Figure 7A) shows similar bands as the spectrum recorded with 647 nm excitation but with an additional band at 1,565 cm^−1^, which is more prominent at pH 7.4 than at pH 5 (Supplementary Figure S7). This feature can be assigned to the 13-*cis* conformation of the retinal chromophore in the blue-shifted M state (Muders et al., 2014) and its changing relative intensity recapitulates the pH-dependent changes of the photocycle kinetics.

Based on the assignment of retinal vibrations by resonance Raman spectroscopy, it becomes evident that the light-induced FTIR difference spectra of *Um*Rh1, representing a steady-state difference between accumulated photointermediates and ground state, will contain different compositions of photocycle intermediates at neutral and acidic pH (Figure 7B). While the M state dominates at pH 7.4 (+1,568 cm^−1^), the presence of a red-shifted intermediate at pH 5 is apparent by the positive band at 1,522 cm^−1^. As the stronger bands at 1,512 cm^−1^ have not been observed in the Raman spectrum of the O state, we assign these bands to the apoprotein. The positive band at 1,183 cm^−1^ is significantly higher in frequency than in *Hs*BR (1,168 cm^−1^) (Zscherp and Heberle, 1997) and is usually assigned to 13-*cis* retinal with protonated Schiff base. However, the O state of the L93A variant of *Hs*BR also exhibited such high frequency (Subramaniam et al., 1991) reflecting some ambiguity in the vibrational assignment.

The C=N stretching vibration of the retinal Schiff base is observed in the Raman spectra at 1,650 cm^−1^ (Supplementary Figure S7) and displays a downshift by 22 cm^−1^ upon H/D exchange (inset of Supplementary Figure S7), which is consistent with a protonated Schiff base. The concomitant band narrowing from 18 to 11 cm^−1^ FWHM indicates the presence of a nearby water molecule (Hildebrandt and Stockburger, 1984).

Based on this assignment, the small negative feature at 1,650 cm^−1^ in the FTIR difference spectrum (Figure 7B) can be assigned to the C=N stretching mode of the retinal Schiff base which is more prominent in the M intermediate at pH 7.4. Instead at pH 5, a prominent band at 1,668 cm^−1^ arises, which can be tentatively assigned to an amide I vibration, indicating protein conformational changes associated with the O intermediate. Bands at 1,761 (+) and 1,737 (−) cm^−1^ are assigned to the C=O stretching modes of aspartic or glutamic acids, indicating a (de-)protonation of these residues (Barth, 2007). Both bands are sensitive to deuteration and show a frequency downshift of about 10 cm^−1^ (Supplementary Figure S8). In the absence of mutational studies, the band at 1,761 cm^−1^ may be tentatively assigned to the protonation of the primary proton acceptor in analogy to *Hs*BR (1,762 cm^−1^ D85 in *Hs*BR) (Radu et al., 2009). Similarly, the negative band located at 1,737 cm^−1^ may indicate the deprotonation of the proton donor to the Schiff base (1,741 cm^−1^ D96 in *Hs*BR) (Radu et al., 2009). However, the fact that the relative band intensities of the bands at 1,761 and 1,737 cm^−1^ barely change upon acidification might oppose this assignment. In *Hs*BR, the pK_a_ of D96 drops from >12 in the dark state to 7.1 in the N-intermediate permitting proton transfer to the retinal Schiff base. Therefore, the negative band assigned to D96 vanishes at pH < 7 (Zscherp et al., 1999).

## Discussion

*Ustilago maydis* comprises three microbial rhodopsin genes Um*ops1*, Um*ops2* and Um*ops3* (Estrada et al., 2009). We investigated the translational product of the former*,* which we name here *Um*Rh1 and which fits to the classification of Brown and Jung (Brown and Jung, 2006) to be a *Hs*BR-like proton pump. For further, more detailed classifications see (Adam et al., 2018) (Wang et al., 2018). Cells of *Pichia pastoris* can heterologously express large amounts of *Um*Rh1 turning the cells red after induction and retinal supply.

**FIGURE 8:**
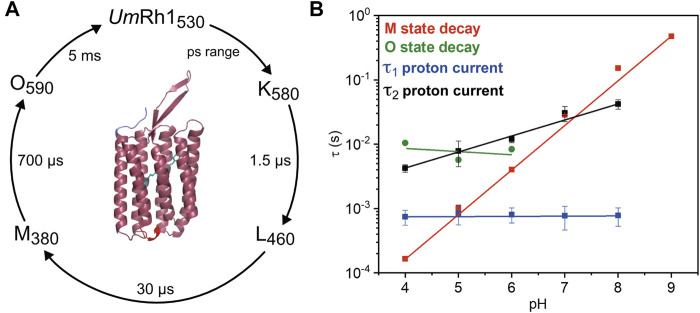
Photocycle of solubilized *Um*Rh1. **(A)** The depicted photocycle plot describes the intermediate state transitions of *Um*Rh1 in 100 mM NaCl, 50 mM sodium acetate, pH 5, 0.03% DDM. The numbers show the maximal difference absorption wavelength of each intermediate state and the corresponding time constant. In the center, a homology model of *Um*Rh1 is shown by using the fungal rhodopsin LR (pdb: 7bmh) (Zabelskii et al., 2021) as template. The retinal (green) is imported from structure of *Acetabularia* Rhodopsin I (pdb: 5awz). The N and C termini are indicated with blue and red. **(B)** pH dependence of the M (red squares) and O state decays (green spheres) and the kinetics of proton translocation as derived from electrophysiology. Data points relate to the two time constants of the decay kinetics of the proton currents (τ_1_ in blue and τ_2_ in black squares, see Table 1 for the values). Straight lines are guides to the eye.

Our studies on the proton-pumping activity of live *Pichia* cells indicated that the expressed protein is functional to pump protons out of the cell, thereby acidifying the surrounding solution. In patch clamp studies on NG108-15 cells, strong pH dependence of proton pumping was assessed with highest current amplitudes and fastest kinetics in the acidic pH range. The absorption maximum of the retinal chromophore at 530 nm, which is only little influenced by the change in pH and titration experiments, revealed a protonatable amino acid with a pKa of around 5. The pKa of the retinal Schiff base of *Um*Rh1 was determined to be 10. Based on our time-resolved UV/Vis spectroscopic results, we suggest a photocycle model of *Um*Rh1 comprising the sequence of K, L, M and O states (Figure 8A). This nomenclature is derived from *Hs*BR (Lanyi, 2006) and we suspect that the rise of the photocycle intermediates relate to different steps in proton translocation across *Um*Rh1 accompanied by protonation and deprotonation events of individual carboxylic side chains of Asp and Glu amino acids and the retinal Schiff base. The O intermediate of *Um*Rh1 is not present at alkaline pH, which agrees with *Hs*BR under alkaline conditions. The N intermediate appears in *Hs*BR at elevated pH but no evidence for its presence was found in *Um*Rh1.

Proton translocation as derived from electrophysiological experiments (Figure 5) is characterized by two time constants. The faster time constant τ_1_ is pH-independent (blue squares in Figure 8B) but the slower process τ_2_ (black squares in Figure 8B) correlates with the slowest kinetics of the photoreaction which is the decay of the O state (green spheres in Figure 8B) at acidic pH and the decay of M at neutral and alkaline pH (red squares in Figure 8B).

The lifetime of the M state of *Um*Rh1 is extended with increasing pH and follows a linear correspondence with the proton concentration (red squares in Figure 8B). Such observation argues in favor of direct reprotonation of the retinal Schiff base from the aqueous bulk solution. The D96N variant of *Hs*BR, in which the internal proton donor is absent, exhibits very similar kinetic behavior (Tittor et al., 1989) (Holz et al., 1989). Thus, we infer that the proton-pumping rate of *Um*Rh1 is not controlled by an internal proton donor. This is a surprising fact considering that the homologous residue of D96 in *Hs*BR is E129 in *Um*Rh1, as derived from sequence alignment (Supplementary Figure S9) (Panzer et al., 2019). Of particular note is the fact that this glutamate/glutamic acid is conserved in the homologous *Neurospora* rhodopsin which does not pump protons (Brown et al., 2001). The FTIR difference spectra (Figure 7B) reveal a negative band at 1,737 cm^−1^ which may be tentatively assigned to the C=O stretching vibration of protonated E129 of *Um*Rh1 whose absorption change is identical at pH 7.4 and pH 5. In *Hs*BR, this difference band disappears at acidic pH due to fast reprotonation of D96 (Zscherp et al., 1999). Thus, E129 of *Um*Rh1 may not act as internal proton donor to the retinal Schiff base. This conclusion will be scrutinized by future FTIR difference experiments on E129 variants.

Overall, the photocycle kinetics of *Um*Rh1 at pH 7.4 is slightly slower than in *Hs*BR but strong acceleration of the M to O conversion was recorded for *Um*Rh1 at acidic pH. A similar acceleration has been reported for PhaeoRD2, a rhodopsin from *Phaeosphaeria nodorum* (Fan et al., 2011). Acceleration of the *Um*Rh1 photocycle is also observed in the presence of the plant hormone IAA. This finding is reminiscent to the acceleration of the reprotonation of the retinal Schiff base by weak acids, like azide, cyanate, nitrite, formate and acetate, which is limited by proton concentration in D96 variants of *Hs*BR (Tittor et al., 1989).

Despite some striking functional and structural similarities of *Um*Rh1 and *Hs*BR, there are marked differences regarding their respective biological role. In halobacteria, *Hs*BR converts light energy to generate a proton gradient for downstream formation of ATP by the respective synthase. We note that *Hs*BR is synthesized by halobacteria only under phototropic conditions, i.e. under environmental stress of low oxygen tension when respiration ceases. As *Um*Rh1 was found in the plasma membrane of the fungus (Panzer et al., 2019) and eukaryotes do not possess such ATP synthases, we infer that the role of *Um*Rh1 is not related to energy conversion. We can only speculate why the fungus in *Ustilago maydis* acidifies its neighboring medium. Fungi in general use proton motive force for their supply with nutrients. Its maintenance is associated with ATP consumption, which probably can be circumvented by using light for proton extrusion. Further, as there is a clear effect of IAA on the *Um*Rh1 activity as was shown in this work and others (Panzer et al., 2019), the involvement in the pathogenic state of *Ustilago maydis* on maize plants via its three different rhodopsins must be considered. Although we have no direct prove that *Um*Rh1 is part of the infection process since we have studied only the isolated rhodopsin, we can speculate on its potential role in the mechanism of pathogenesis. Of particular note is the fact that while *Um*Rh1 being localized in the plasma membrane, but not the second proton pump, is facing potential interaction sites of the fungal cells to the environment, which is the host plant during infection. It remains unclear if acidification of the environment, which is triggered by light, transfers a signal to the fungus being neither inside the plant nor in the ground, helps to infect its target. It has been suggested (Sanchez-Arreguin et al., 2021) that NRG1 of *Ustilago maydis* is changing the expression of 368 genes in response to acidification, many of them being virulence factors. The expression of *Um*Rh1 is induced by illumination of Wco1 with the expression of Wco1 being induced by low pH via NRG1. Finally, *Um*Rh1 generation in the fungus is also dependent on acidic conditions. As an outward directed proton pump, *Um*Rh1 acidifies the environment triggering its own expression as well as the expression of other virulence factors. In addition, low pH influences the stage within the dimorphism of the fungus triggering the transition between haploid or yeast like growth and filamentous diploid or mycelial growth in pathogenesis (Martinez-Espinoza et al., 2004), which is regulated on the DNA level by the homeodomain transcription factors bEast and bWest (Lanver et al., 2017) (Schulz et al., 1990). In *Neurospora crassa* a knockout variant of the rhodopsin gene for NOP-1 was unable to undergo the asexual-sexual transition, indicating rhodopsin´s role in this switching process (Wang et al., 2018). With respect to *Um*Rh1, the pH-dependent dimorphism switch would be facilitated in light as the rhodopsin triggers the acidification in a light-dependent manner. As two publications exclude the fungal rhodopsins and the IAA being the single key of triggering pathogenesis (Estrada and Avalos, 2009) (Reineke et al., 2008), both factors have not been tested simultaneously in a double variant so far, as each of them can lead to acidification redundantly and both factors influence each other leading to even larger decrease in pH. It is not clear if the infection process is modulated by these factors at all. Here, one may take a closer look at tumor outgrowth comparing the fungal double variant to the wild-type strain. Further investigations are necessary to clarify these points, as we hypothesize the fungal rhodopsins may have an impact on pathogenesis which could not be clearly elucidated by experiments due to redundant mechanisms. We infer that such alternate mechanisms may help to adapt to different conditions and provide the fungus with an evolutionary advantage.

## Data Availability

The original contributions presented in the study are included in the article/Supplementary Material, further inquiries can be directed to the corresponding author.
