# Supplementary material for: The Photoreaction of the Proton-Pumping Rhodopsin 1 From the Maize Pathogenic Basidiomycete Ustilago maydis
Source: Front Mol Biosci. 2022 Feb 25;9:826990. doi: 10.3389/fmolb.2022.826990 (PMC8913941; doi:10.3389/fmolb.2022.826990)
Supplement: Supplementary file 1 [file DataSheet1.docx]

**SUPPLEMENTARY INFORMATIONS**

**
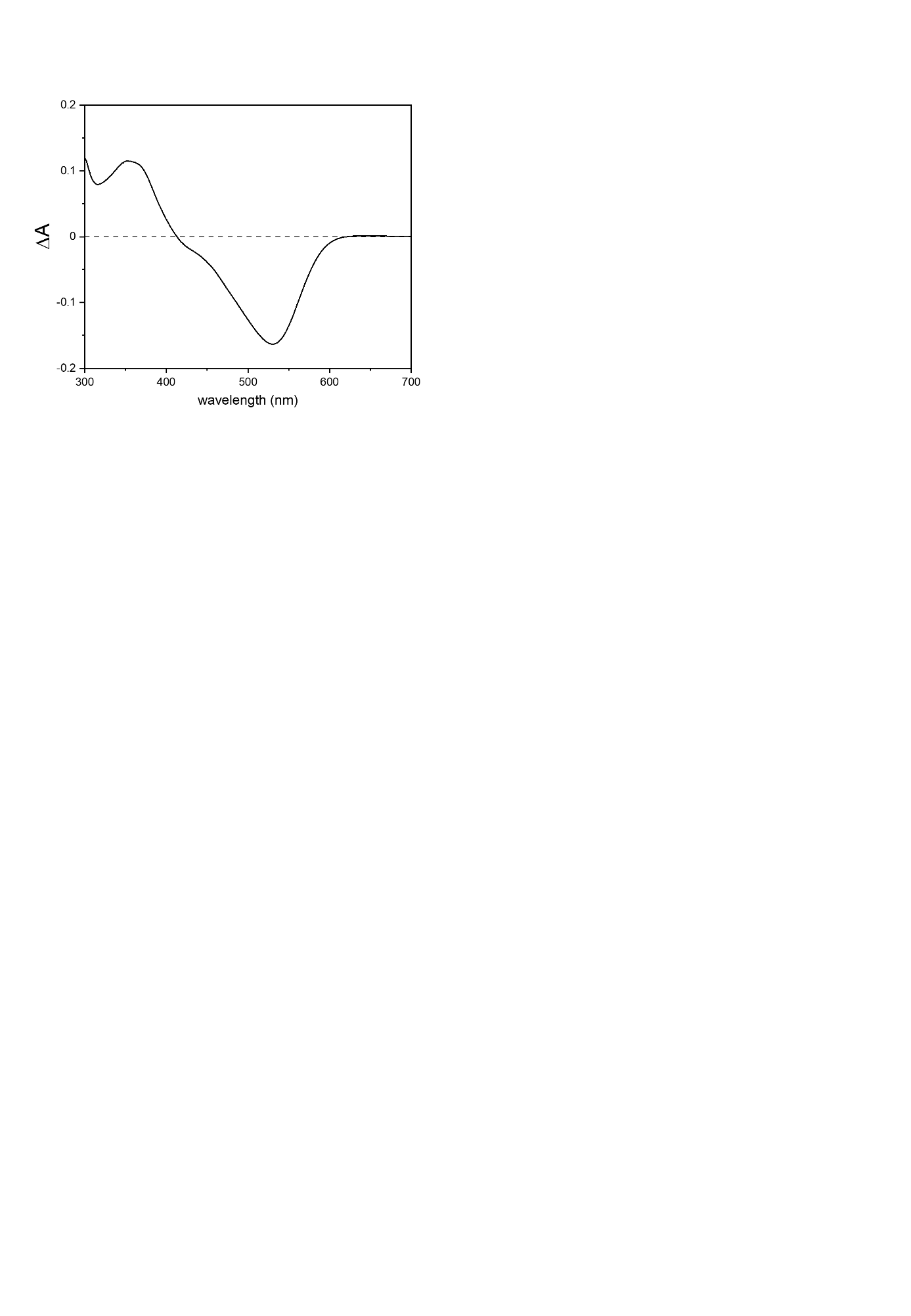
**

**Figure S1. Difference UV/Vis spectrum for extinction coefficient calculation.** Difference spectrum before and after addition of 10 mM hydroxylamine to *Um*Rh1 is shown. The values indicate the absorption peaks of retinal oxime (ΔA_352_ = 0.115) and retinal in native *Um*Rh1 (ΔA_531_ = -0.164).

**
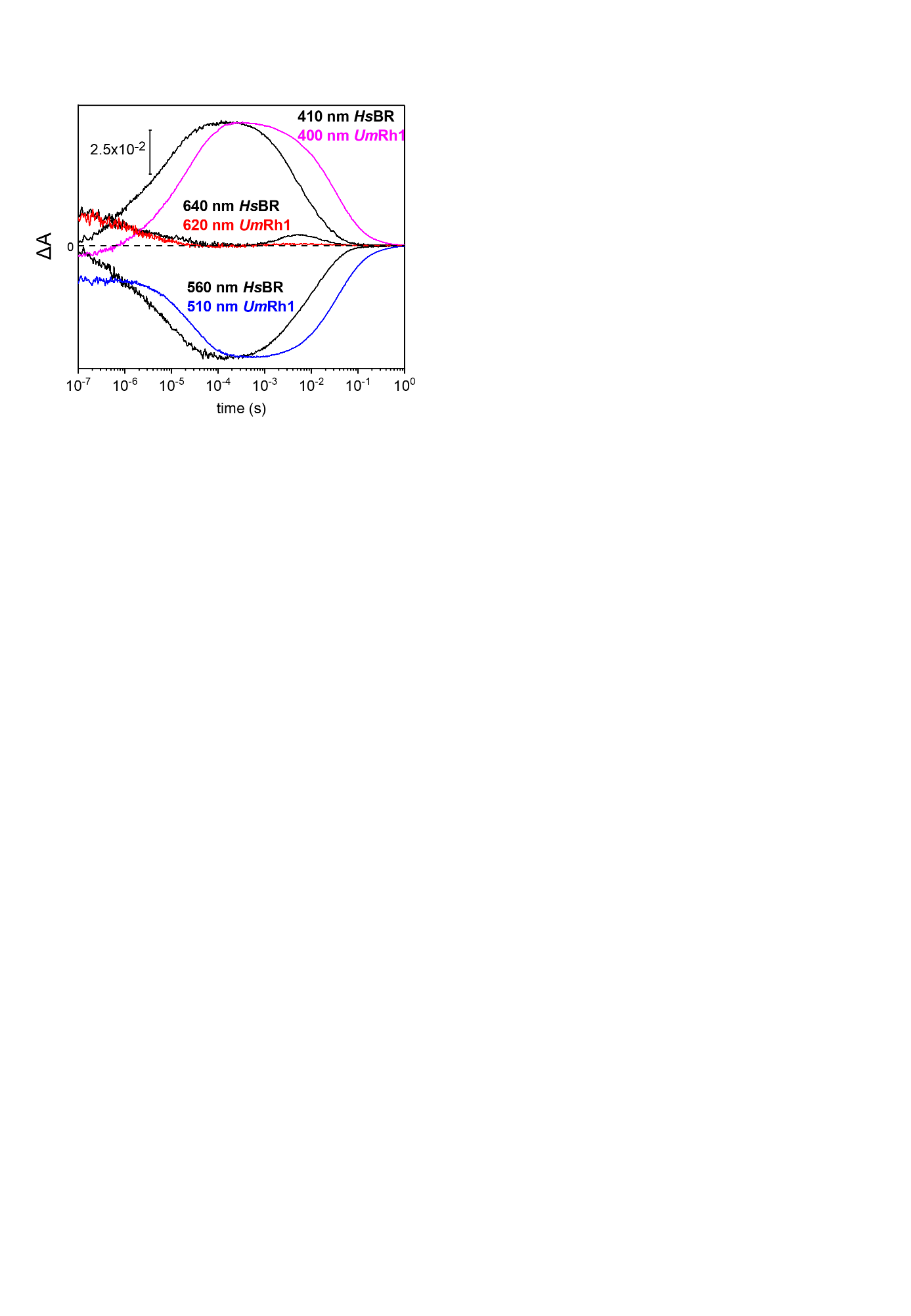
**

**Figure S2. Kinetics comparison between *Um*Rh1 and *Hs*BR at pH 7.4.** Time evolution of the flash-induced absorption changes of *Um*Rh1 and *Hs*BR under the same buffer conditions (100 mM NaCl, 20 mM HEPES, pH 7.4 and 0.03% DDM) is shown. The chosen wavelengths for *Um*Rh1 are 400 nm, 510 nm, and 620 nm, while for *Hs*BR are 410 nm, 560 nm, and 640 nm, corresponding to M, ground state and O state, respectively.

**
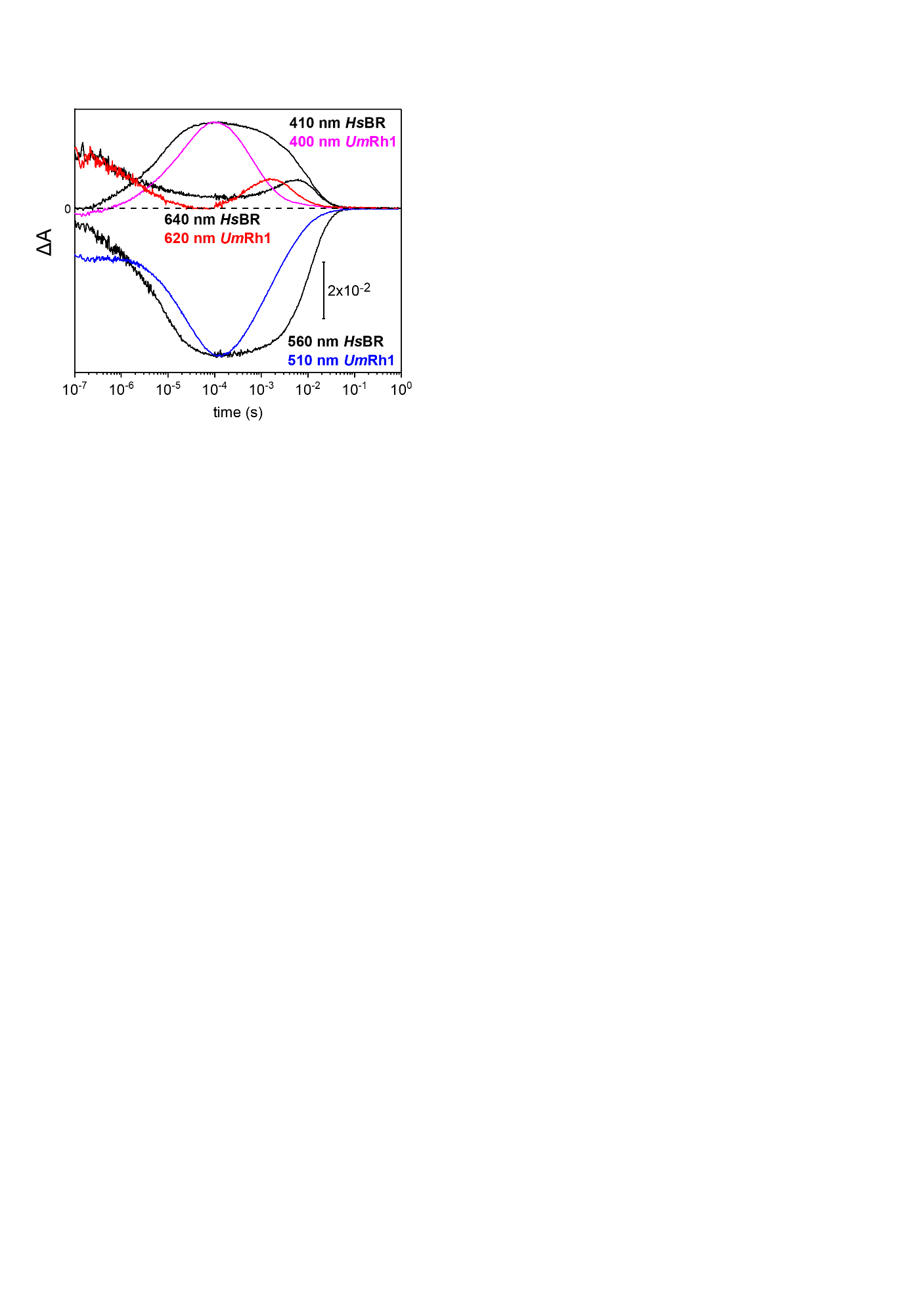
**

**Figure S3. Kinetics comparison between *Um*Rh1 and *Hs*BR at pH 5.** Time evolution of the flash-induced absorption changes of *Um*Rh1 and *Hs*BR under the same buffer conditions (100 mM NaCl, 50 mM buffer mix (10 mM HEPES, MOPS, MES, citrate and glycine), pH 5 and 0.03% DDM) is shown. The chosen wavelengths for *Um*Rh1 are 400 nm, 510 nm, and 620 nm, while for *Hs*BR are 410 nm, 560 nm, and 640 nm that represent the M state, ground state and O state.

**
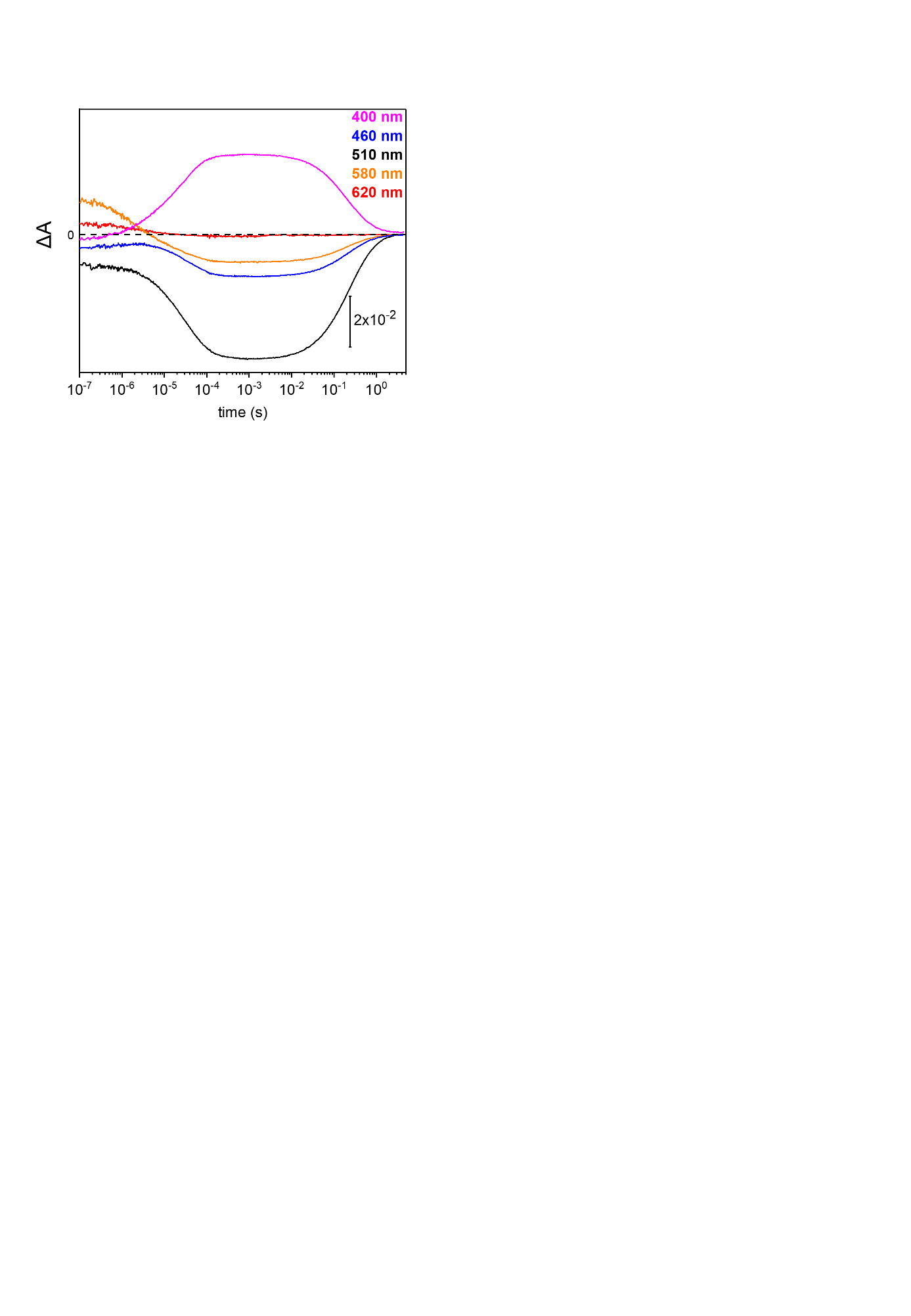
**

**Figure S4. Photoreaction kinetics of *Um*Rh1 reconstituted in nanodiscs at pH 7.4.** Time evolution of the flash-induced absorption changes of the *Um*Rh1 reconstituted in nanodisc (MSP1D1-DMPC) in 100 mM NaCl, 20 mM HEPES, pH 7.4 is shown. The chosen wavelengths are 400 nm, 460 nm, 510 nm, 580 nm and 620 nm that are representative of M, L, ground state, K and O state respectively.

**
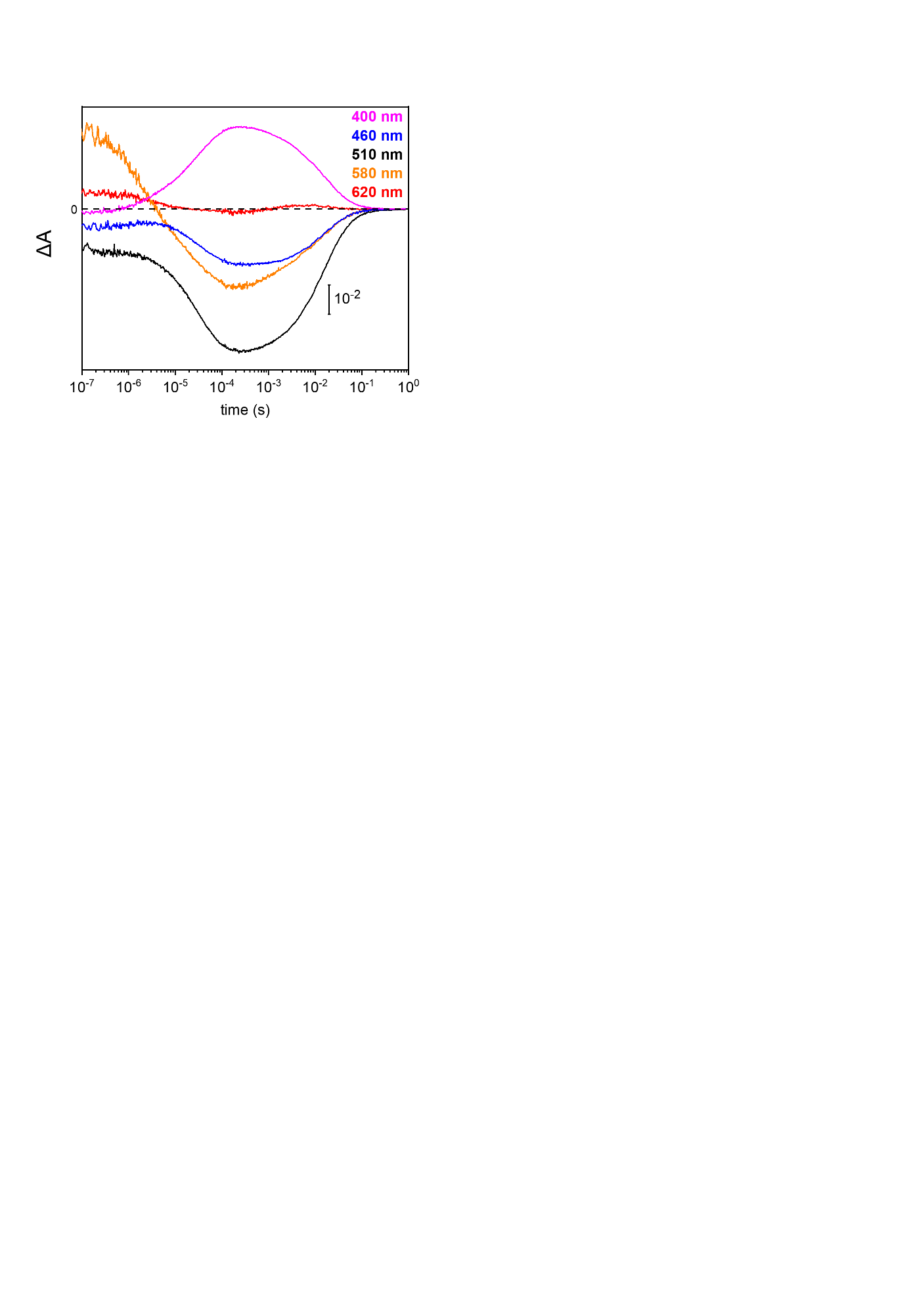
**

**Figure S5. Photoreaction kinetics of *Um*Rh1 reconstituted in nanodiscs at pH 5.** Time evolution of the flash-induced absorption changes of the *Um*Rh1 reconstituted in nanodisc (MSP1D1-DMPC in 100 mM NaCl, 50 mM buffer mix (10 mM HEPES, MOPS, MES, citrate and glycine), pH 5 is shown. The chosen wavelengths are 400 nm, 460 nm, 510 nm, 580 nm and 620 nm that are representative of M, L, ground state, K and O state respectively. The O state, monitored at 620 nm, is still remarkable, in comparison to the O state kinetic recorded at neutral pH.

**
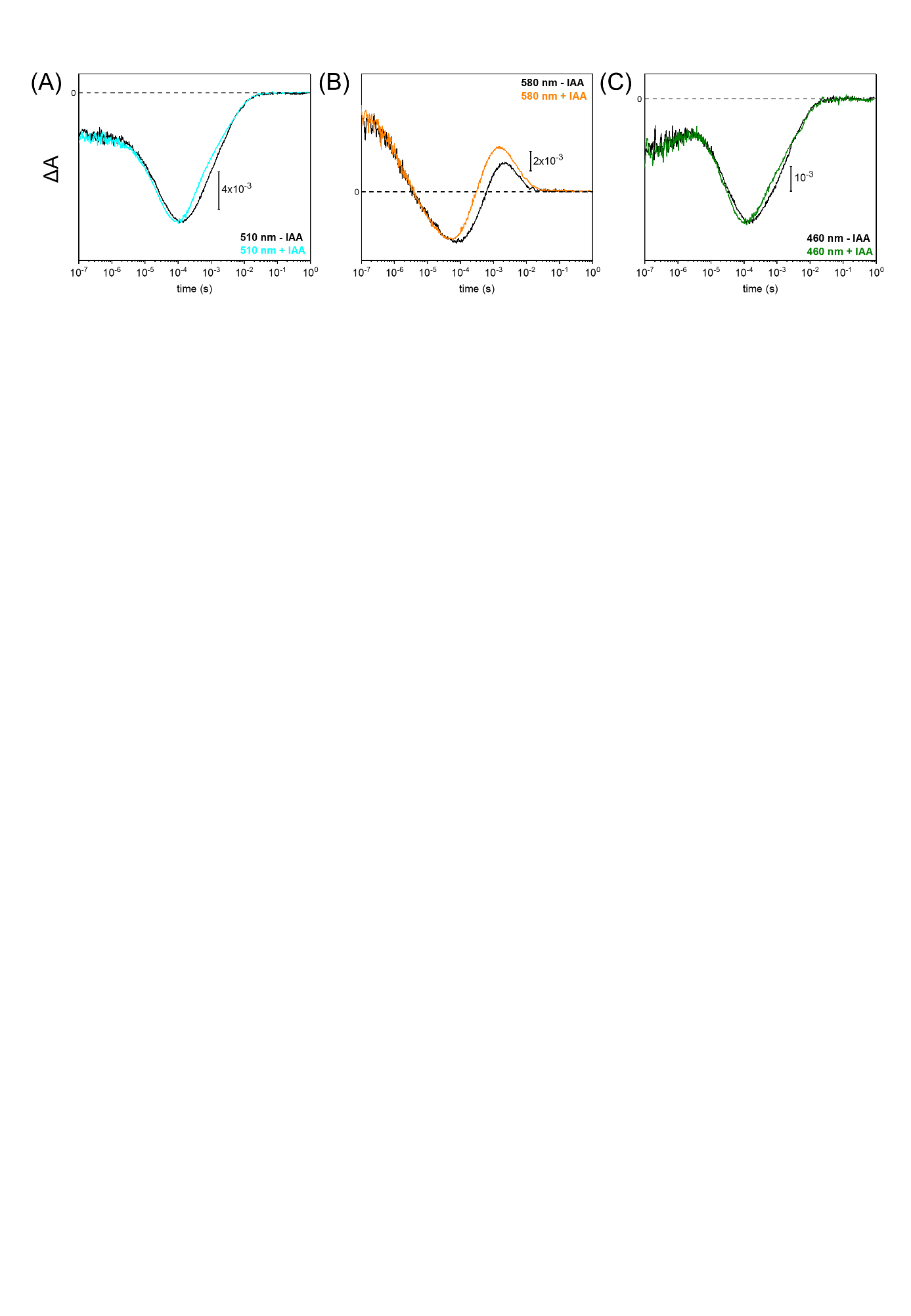
Figure S6. Photoreaction kinetics of *Um*Rh1 in presence and absence of IAA.** Time evolution of the flash-induced absorption changes of the DDM-solubilized *Um*Rh1 (100 mM NaCl, 100 mM MES, pH 5.5 and 0.03% DDM) and DDM-solubilized *Um*Rh1 in presence of 20 mM IAA (in the same buffer) is shown. The chosen wavelengths are 510 nm panel **(A)**, 580 nm panel **(B)** and 460 nm panel **(C),** representative of the ground state bleaching, K state and L state respectively.


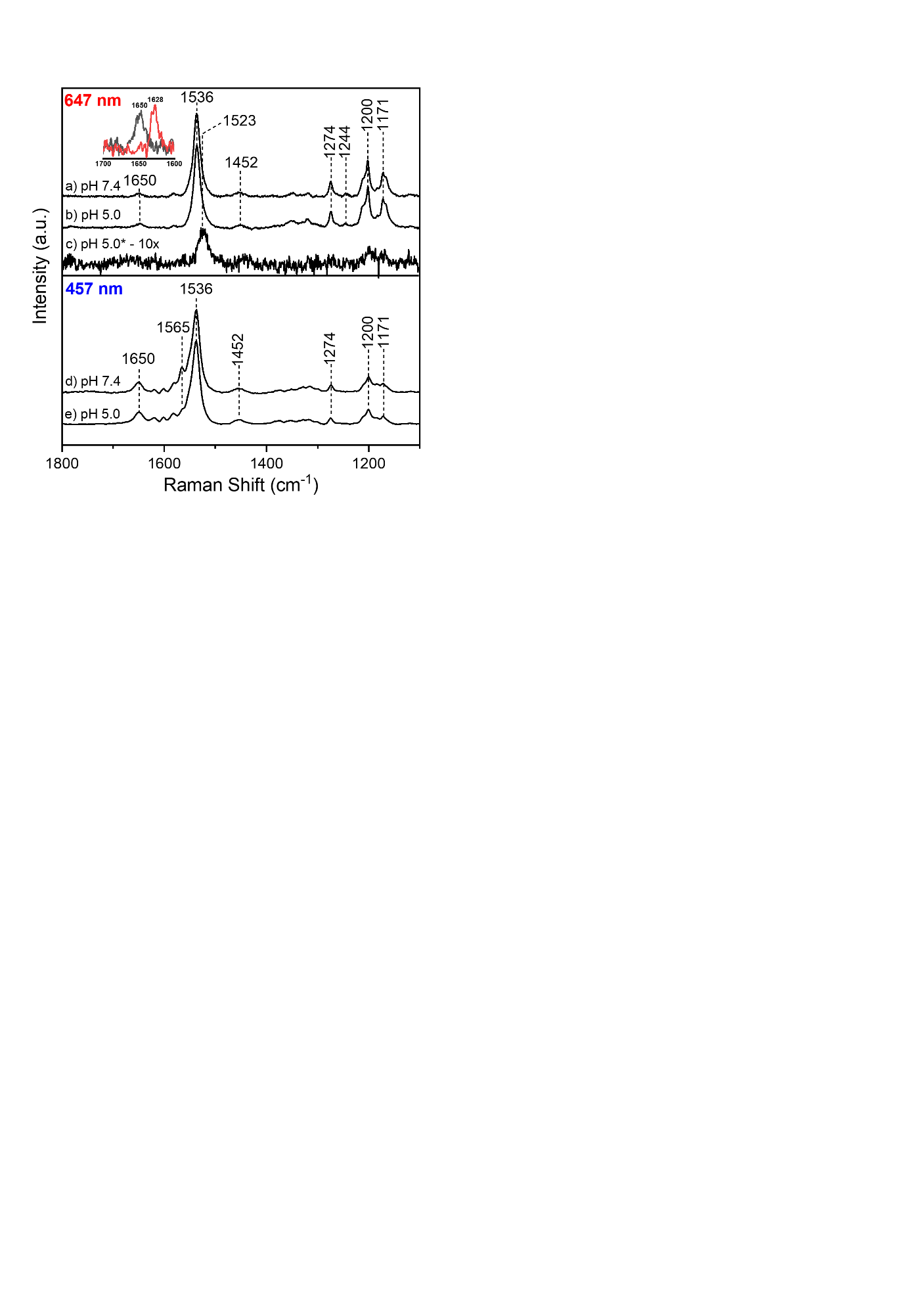


Figure S7. Resonance Raman spectra of *Um*Rh1. The spectra were recorded at 647 nm (a, b, c) and 457 nm (d, e) excitation at pH 7.4 and pH 5, as indicated. The inset in the top left corner shows the H/D exchange experienced by the Schiff base C=N stretch in the range between 1700 and 1600 cm^-1^ (black – H_2_O; red – D_2_O). Spectrum c is the difference spectrum due to additional illumination at 530 nm at pH 5.

**
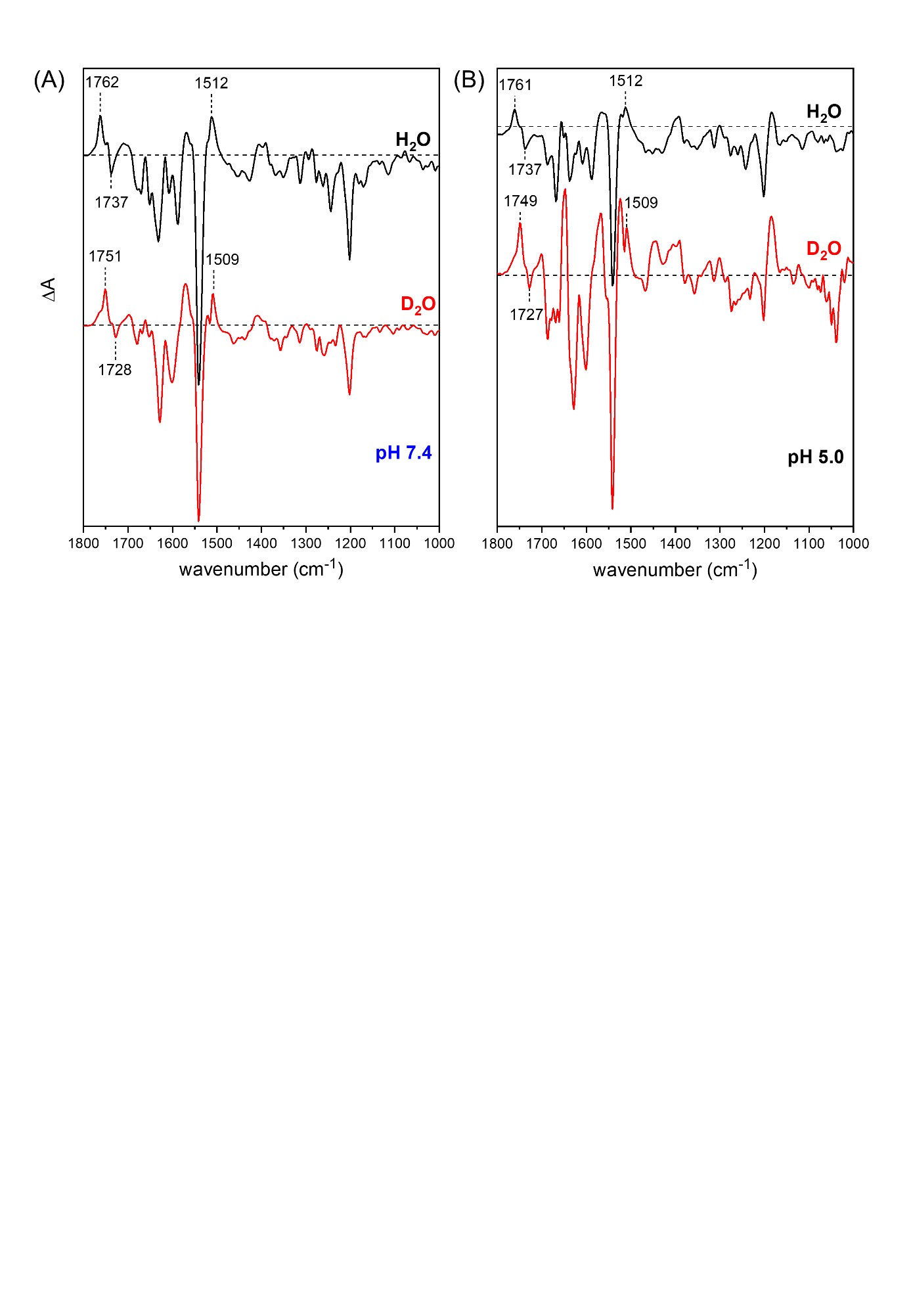
**

**Figure S8. FTIR spectra at pH 7.4 (A) and pH 5 (B) in H_2_O (black) and D_2_O (red).**


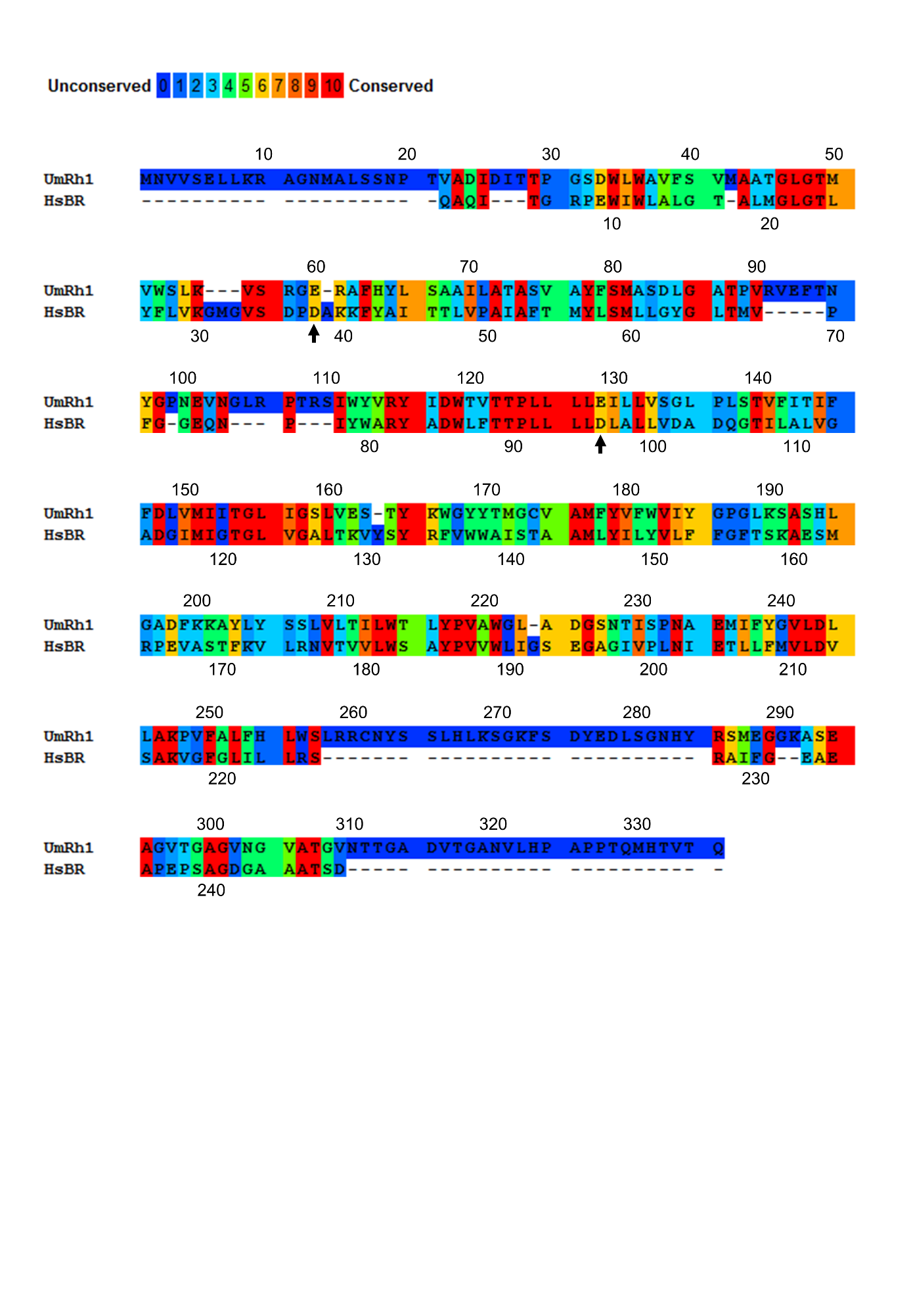


Figure S9. Multiple sequence alignment of *Um*Rh1 and *Hs*BR. The numbers correspond to the amino acid residues of *Um*Rh1 and *Hs*BR, respectively. The signal peptide of 13 amino acids at the N-terminus of *Hs*BR is deleted here to match the numbering in the literature. The residues discussed are indicated by black arrows. This alignment was done by the PRALINE program, an online resource of Centre for Integrative Bioinformatics VU, Vrije Universiteit Amsterdam.
